# Camouflaging Autism in Pursuit of Friendship and Intimate Relationships: A Systematic Review

**DOI:** 10.1089/aut.2023.0160

**Published:** 2025-10-24

**Authors:** Kathryn Ridgway, Kassandrah Cooke, David H. Demmer, Merrilyn Hooley, Elizabeth Westrupp, Mark A. Stokes

**Affiliations:** School of Psychology, Healthy Autistic Life Lab, Deakin University, Geelong, Australia.

**Keywords:** autism, camouflaging, friendship, social motivation

## Abstract

**Background:**

Difficulties in making and maintaining social relationships is common among autistic people. These difficulties are thought to be due to differences in autistic social interaction and social communication that have been theorized to be the result of reduced social motivation. We argue that this theory does not account for camouflaging, a combination of strategies that are employed frequently by many autistic individuals in pursuit of obtaining social relationships; however, its utility in achieving satisfactory friendship and intimate relationships for the individual is not clear.

**Method:**

This review synthesizes empirical evidence of camouflaging as a strategy for achieving friendships and/or intimate relationships among autistic adolescents and adults. A systematic search yielded 1421 records. Forty-nine full texts were screened, and eight qualitative studies were retained.

**Results:**

Results reveal that camouflaging was a main strategy in pursuit of friendship, with little evidence for intimate relationships, in this sample of autistic adolescents and adults. For most, camouflaging is exhausting, often ineffective and inefficient, and ultimately a challenge to one’s social authenticity.

**Discussion/Conclusion:**

Research suggests that many autistic individuals rely on camouflaging in pursuit of friendship and intimate relationships, although this is often a nonpreferred strategy. Some autistic individuals seek to replace camouflage with alternative strategies to obtain social relationships, yet outcomes typically remain dissatisfying. This demonstrates that we need to reconsider the social motivation theory, and to better understand alternative strategies that can help autistic individuals to obtain authentic, satisfactory social relationships, and to support opportunities to achieve these desired outcomes.

**Community Brief:**

**Why is this topic important?**

Many autistic individuals feel dissatisfied with the quality and quantity of their social relationships and are, therefore, more vulnerable to loneliness and reduced well-being. This may be because autistic people communicate and interact differently to non-autistic individuals. These differences can, at times, be misinterpreted as a lack of motivation to socialize and connect with other people. But many autistic individuals are motivated to build meaningful friendships and intimate relationships and often rely on camouflaging to achieve these outcomes. Regardless of their efforts, many autistic people continue to struggle to make the social connections they desire. Therefore, the effectiveness of camouflaging on building friendships and intimate relationships needs to be better understood.

**What was the purpose of this review?**

We set out to understand the impact that camouflaging has the friendships and intimate relationships of autistic individuals by examining the existing research.

**What are the results of this review?**

We found that camouflaging is a common strategy used by many autistic individuals to connect with others in aim to build and maintain friendships, especially with non-autistic people. Pressure to camouflage began early in life, for many autistic participants, and continued to increase in both complexity and use across the lifespan. However, many autistic individuals remained dissatisfied with the friendships and intimate relationships gained through its use. For many, camouflaging prevented genuine social interactions, and the friendships that resulted from its use were often lacking in closeness and authenticity for the autistic individual. To reduce the reliance on camouflaging, some autistic individuals socially withdrew, or attempted to find friendship within the autistic community or in social contexts where camouflaging felt less necessary. Yet, many autistic participants remained feeling lonely, frustrated, and dissatisfied with their social relationships.

**What were the weaknesses of this review?**

Only half of the eight studies involved an autistic researcher and author, while most studies incorporated community participation in the design of the study. We found that participants were mostly female, from countries such as the United Kingdom and United States of America, and most participants were above18 years of age. Results may be impacted by a lack of researcher and sample diversity, and minimal autistic input in study design and interpretation; thus, generalization of these findings may be limited. Camouflaging is an evolving area of research, and we recognize that there may be terminology or perspectives that were not captured by our review.

**What do the authors recommend and how will this help autistic adults in the future?**

More research is needed to better understand autistic social interactions and to support alternative strategies to camouflaging, including environmental modifications and changes in the way non-autistic people understand and interact with autistic people. We recommend that autistic individuals’ strengths and needs drive this area of research, and that supports address ways that all people can help to enhance autistic social outcomes. We anticipate that these strategies will promote more authentic social relationships, reduce reliance on camouflage, help to decrease loneliness, and improve well-being among autistic individuals.

Autism Spectrum Disorder (hereafter autism) is a lifelong neurodevelopmental condition.^1^ The diagnostic rates of autism are increasing, and based on formal diagnostic data, the current prevalence rates are between 1% and 1.68% worldwide,^1,2^ with rates as high as 2.78% reported in the United States of America.^3^ Autism is characterized by challenges in social interaction and social communication, as well as restrictive, repetitive patterns of behavior, with many autistic individuals^1^ also experiencing hyper- and/or hyposensitivity to sensory information.^4^ These characteristics present heterogeneously and often vary for individuals across the lifespan,^7,8^ and can include strengths such as logical thinking, honesty, trustworthiness,^9^ and creativity.^10^

Regardless of an individual’s experience of autism, their social lives are significantly impacted, especially when the demands of a social environment exceed the non-autistic social skills or social preferences of the individual.^11^ These difficulties are often exacerbated by ineffective support,^12^ barriers to social inclusion,^13^ such as stigma, discrimination, and a lack of autism awareness that is common in many social environments.^14,15^ Unfortunately, for many autistic individuals, these social difficulties can limit social participation,^16,17^ and effect the ability to form and maintain social relationships; thus, they may develop fewer friendships compared with non-autistic individuals.^18,19^ Those who are less satisfied with the quantity and quality of their social relationships have been shown to experience negative psychological outcomes, including loneliness,^20^ isolation,^21^ reduced well-being,^22^ anxiety,^23^ depression,^24^ suicidal ideation, suicide attempts, and suicide completion.^13^ Thus, urgent attention is required to enhance the social relationships desired by autistic people.

Social Motivation theory (SMt) posits that autistic individuals likely have decreased social motivation, negatively impacting on social connection.^25^ According to SMt, autistic individuals do not attend to important social information and have reduced sensitivity to social reward, both of which are thought necessary to develop the social skills considered important for proficient social interactions.^25^ However, there is overwhelming evidence that many autistic individuals actively pursue social relationships, engage with others,^9,10^ find social interactions rewarding,^26^ desire meaningful friendships,^19^ romantic relationships,^27,28^ and want to participate within the community.^17,29^ Despite these desires, many autistic individuals report feeling defeated by a lack of confidence in their social skills,^30^ negative social experiences, and remain dissatisfied with their social relationships.^31–33^ Repeated negative social experiences can lead to social withdrawal,^31,32^ which serves to exacerbate already limited social opportunities,^34^ and can further weaken the well-being of many autistic individuals.^20^

These adverse social and psychological outcomes are commonly attributed to the autistic individual’s social differences, because it is evident that many autistic people have difficulty negotiating social interactions with non-autistic people.^18,35^ Despite these challenges, many autistic individuals value friendship qualities similar to those identified by non-autistic people, including reciprocity, trust, respect, shared interests, and proximity.^36,37^ Furthermore, autistic individuals seek companionship, affection, mutual support, happiness, and shared experience with others.^38^ The strategies used to attain these types of connections may appear to be different, but are no less important, valuable, or satisfactory for autistic people when obtained.^18,38^ However, to achieve these outcomes, the pressure falls heavily on the autistic individual to modify their social behavior rather than on non-autistic people, demonstrating a disparity in responsibility between autistic and non-autistic neurotypes.^39^ Thus, we suggest that SMt is built on non-autistic understandings of social connection and it fails to recognize the developmental trajectories and social differences common in autism as evidence of social motivation.^35^

Formal social skills training programs are commonly offered to help address the social difficulties experienced by autistic individuals.^40^ Such interventions are focused on modifying autistic social behavior by teaching non-autistic social rules with the aim to improve social engagement and to help participants build friendships.^41–43^ Although well-intentioned, participation in social skills training can, at times, reinforce the belief that an individual’s own social behaviors need to be suppressed and replaced.^12,44^ The newly learned social skills can then become part of an individual’s *camouflaging* strategies (also known as *masking, compensation, passing as normal,* and *adaptative morphing*),^45–47^ which may signal that one’s autistic characteristics are inadequate and likely contributes negatively to self-perception and the interpersonal trauma experienced by many autistic individuals.^12,45,48^ Even for those autistic people who seek support to improve their ability to interact with others, reveal that available programs are often ineffective and fail to target the priorities of the autistic individual.^12^

Camouflaging is used by many autistic individuals in an attempt to minimize autistic characteristics and to appear more like non-autistic people when interacting with others, especially non-autistic individuals.^46,49^ These strategies are most common among those who are more attuned to their social difficulties and seek to resolve them.^50,51^ In addition to the social rules that are learned via formalized programs,^43^ camouflaging often involves conscious and unconscious mimicry of observed social behavior that are assumed by an autistic individual to be necessary for successful social interaction.^46,52,53^ Its use is often encouraged by non-autistic people who are close to an autistic individual and seek to strengthen relational communication and connection, both by teaching non-autistic social skills and reinforcing their use, which may lead to increased reliance on camouflaging.^54^ Camouflaging has been likened to impression management strategies, which are employed by most people to overcome communication difficulties and to influence how others perceive them in an attempt to maintain a positive social status.^55,56^ However, autistic camouflaging appears to differ from common impression management strategies, as there appears to be a greater disparity between an autistic individual’s natural social communication and behavior, and the way in which they interact in the world when camouflaging; often with significant impact on the individual’s identity and well-being.^51,52^

Camouflaging incorporates a variety of concurrent strategies, including observing, imitating, and practicing others’ social behaviors,^57,58^ minimizing repetitive movements and personal interests, using conversation scripts,^46,59^ and minimizing sensory distress and emotional responses.^51^ Beginning in early life, camouflage appears to be gradual and adaptive response to difficulties in understanding and meeting non-autistic social expectations,^44,60^ social rejection, bullying and victimization,^53,54^ explicit teaching,^61^ and dissatisfied social desires.^61^ The use of these strategies vary in complexity and intensity based on the social context in which they are occurring, the social partners present, and on the autistic individual.^55^ As such, at any given time, autistic individuals may experience varying degrees of social motivation, need for self-preservation,^62^ or felt social pressure, which may be reflected in the type and intensity of camouflaging that is employed.^54,55,62^

Camouflaging can, at times, increase peer acceptance,^63^ avoid negative perceptions and negative experiences,^64^ increase likability,^65^ and help to build friendships and intimate relationships.^53,66^ These positive outcomes likely motivate the continued use of camouflaging; however, camouflaging is exhausting,^59^ may be unreliable in more complex social interactions, can be anxiety provoking,^53,67^ stigmatizing, unsustainable,^52,68^ and may not achieve desired, genuine social relationships for many autistic people.^51,54,69^ Furthermore, camouflaging can lead to feelings of confusion about one’s sense of identity, and a loss of a feeling of authenticity in how they relate with others, which may create a sense of distance between the individual and others they seek to connect with, particularly with non-autistic social partners.^46,59^

The operationalization and measurement of camouflaging and the population characteristics that contribute to variability in strategies and use are an ongoing focus in autism research. Most recent evidence reveals that autistic females are more likely to report camouflaging, especially females who score higher on self-report autism measures, such as the Autism Quotient, although this may be a consequence of the overrepresentation of females in camouflage studies.^47,70^ Cognitive profiles associated with camouflage are lesser understood, which is unsurprising given that autistic individuals with lower IQ (i.e., <70) or who use nonverbal communication, are commonly excluded from camouflage studies.^47^ It is clear that camouflaging is a widely used strategy, yet whether it helps to achieve friendships and intimate relationships that are satisfactory to the individual’s desires and needs remains largely unknown and requires further investigation.

## Purpose and Aim of the Current Review

Our aim is to help evidence camouflaging as a strategy that is used by autistic individuals who are motivated to develop friendships and intimate relationships. It is anticipated that this will help to better understand the impact of modifying autistic socialization to enhance social interactions for those motivated to obtain these social connections. We will be synthesizing evidence that investigates the friendships and intimate relationships that are achieved by autistic adolescents and adults who use camouflaging in pursuit of these social outcomes within general social environments. We sought to answer two questions: (1) Does existing evidence support camouflaging as an effective (i.e., how well does it work) strategy to acquire friendships and/or intimate relationships among autistic individuals who express the motivation to achieve them? and (2) Is camouflaging perceived to be an efficient (i.e., is it cost-effective) strategy for developing friendships and/or intimate relationships among autistic individuals who express the motivation to achieve them?

## Method

### Search strategy and selection criteria

We searched three databases, Cumulative Index to Nursing and Allied Health Literature (CINAHL), Medical Literature Analysis and Retrieval System Online (MEDLINE) Complete, and PsycInfo between February 2022 and September 2023. The search strategy comprised a combination of search words and synonyms, presented in Table 1, to capture the four main variables of interest: autism, camouflaging, social motivation, and friendships and intimate relationships. Studies were filtered to include those published in English that involved adults and/or adolescents, were peer-reviewed publications, and published from earliest available until September 2023. Search results were uploaded into Rayyan, where duplicates were iteratively identified and manually resolved following the guidelines set out by Preferred Reporting Items for Systematic Reviews and Meta-analysis (PRISMA).^71^

**Table 1.** Search Strategy for Titles and Abstracts

Key	Search terms
Autism AND	autis* OR asperger* OR pervasive developmental disorder
Social connection and social participation AND	social participat* OR social connect* OR social competency OR social interact* OR OR friend* OR social skill* OR lonel* OR isolat* OR intima* OR romantic
Social motivation and camouflaging AND	“compensat* behavio*” OR camouflag* OR masking OR imitat* OR mimic* OR “social withdraw*” OR “reduced social*” OR burnout OR overwhelm* OR “social adjustment” OR “social anhedonia” OR “social motivat*” OR “social reward” OR “social disinterest”
Adult and Adolescent AND	adult* OR adulthood OR adolescen* OR youth* OR boy OR girl

The first and second authors screened titles and abstracts, using the following inclusion criteria, based on evidence presented in the introduction, to find studies where: (1) diagnosed autistic participants, where <10% reported self-diagnosis, (2) data for adolescent and adult participants 10 years old and above,^72^ (3) participants were cognitively able due to the trends in camouflaging research, as indicated by an IQ of ≥70 or those who attended a mainstream school or were enrolled in age-appropriate education or were currently or previously engaged in unsupported employment, or those who were able to participate unsupported in a study’s data collection; (4) collected self-report data; (5) included predictor data for social motivation (e.g., quantitative measure or discussion related to a desire for social relationships), (6) the use of camouflage when making or maintaining friendships and/or intimate relationships; and (7) included outcome data for friendship and/or intimate relationships. Data could relate to previous or current experiences of participants. Case studies, review articles, conference and seminar articles, and gray literature were excluded as the current review sought to demonstrate what is found within the current peer-reviewed literature. Peer-review literature attempts to minimize inaccuracies and bias, which can be valuable to examine the similarities and differences between studies in a systematic review, and produce consistency in the results.^73^ The first author conducted the full-text screening, and further studies were removed for reasons recorded in the PRISMA flow-chart shown in the Figure 1 below.

**FIG. 1.**
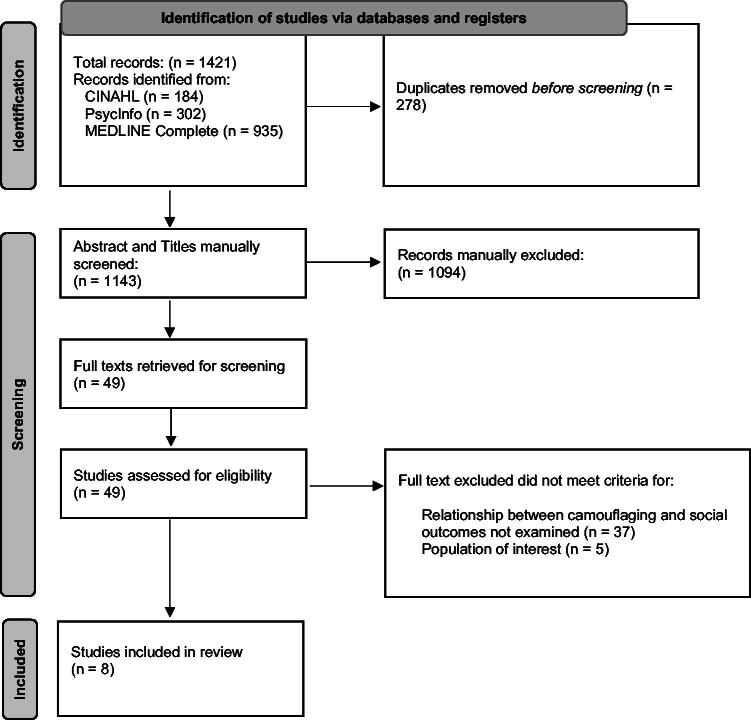
PRISMA flow-chart of systematic search, Adapted from Paige et al.^71^ The figure shows the flow of the search strategy used in the current systematic review. A total of 1421 records were identified after a search of three databases. After 278 duplicate records were removed, 1143 titles and abstracts were screened by the first and second authors. Forty-nine full texts were then carefully screened by the first author. Thirty-seven records did not meet the criteria, as they did not relate to the relationship between camouflaging and the social outcomes of friendship and/or intimate relationships for autistic people seeking these. Five records did not meet criteria for population of interest, based on age or diagnosis. Eight studies were included in the final review. PRISMA, Preferred Reporting Items for Systematic Reviews and Meta-analysis.

### Data extraction and synthesis

Although camouflaging is a commonly used strategy by autistic individuals to enhance social interaction and social communication with others, there were no studies found that specifically measured the friendships and/or intimate relationships that were obtained by study participants. As all of the included studies were qualitative, the first author conducted a thematic synthesis of findings, and MAS and MH contributed to theme development and interpretation.

We used a thematic synthesis approach that was modeled on interpretive synthesis described by Noblit and Hare.^74,75^ Qualitative research reporting relies on an integration of participant respondents into themes that represent the objectives of the study, and the interpretation of participant responses by the authors.^75^ Through this process, we were able to explore associations relevant to the questions asked in a systematic review and also identify related themes that emerged.^74^ A line-by-line screening process was used in this article, which involved:

I Identifying and coding words and phrases in relation to the key terms of this review, including: (1) participants’ previous, current, or future desired friendship and/or intimate relationship experiences, (2) the use of any camouflage strategy when interacting with potential or exiting friends and/or intimate partners, (3) social motivation was inferred by authors as any participants’ current or previous, desire for, or disappointment with, friendship and/or intimate relationships. It was the intent of the current review to examine social motivation as an independent variable; however, an unexpected finding during data extraction was that motivation varied following camouflaging, social interactions, and experiences, demonstrating a multidirectional association between variables. Therefore, social motivation was contextualized within these concepts.
II The relationships between the key terms were then identified and the impact of these associations for autistic participants was also considered.
III The final themes were then developed by synthesizing the most common links between key terms, i.e., occurred within at least 80% of studies, stratified by age.
IV Finally, the themes were contextualized within what is currently known about the key terms among autistic individuals.

In this analysis, we considered the unique experiences of the autistic participants in the context of the interview questions asked while remaining focused on the aims of the review, which allowed for a purposive and integrative synthesis of the literature.^76^

### Quality assessment

The Critical Appraisal Skills Program (CASP) Qualitative Checklist was used to assess the quality of included studies.^77^ This comprehensive tool consists of ten questions that address the appropriateness of several aspects of each study, including the research question, methodology, data collection methods, reporting and interpretation of findings, role of the researcher, ethical considerations, and the value of the research. Each question is assigned either, yes, cannot tell, or no, and responses can be expanded upon by providing additional comments. The first author completed the checklist, and the quality of studies was discussed with two additional authors.

## Results

### Systematic search

As shown in the PRISMA flow chart (see Fig. 1), the initial database searches found 1421 records. Rayyan automatically checked for duplications and further manual screening removed 278 records. The 1101 unique entries were screened by abstract and title by the first and second authors and all conflicts (4.5%) were resolved through discussion; 1094 articles were excluded. Forty-nine full texts were then screened with 41 excluded. Articles were excluded for not exploring the relationship between camouflaging and relevant social outcomes (*n* = 36) or did not meet participant sample criteria (*n* = 5). Eight studies published between 2008 and 2021 were included in the final review.

### Study designs

As shown in Table 2, all eight included studies were qualitative study designs that used novel semi-structured interviews and open-ended questionnaires. Study interviews were conducted in various locations, including the participants’ home or researchers’ home, university conference rooms, and or clinics.

**Table 2.** Studies and Participant Demographics

Study and Country	Participants		% Gender		M *(SD)* [range] age		Confirmation		Community participation	Participant recruitment
	A	NA	A	NA	A	NA	Cog	A		
Müller et al. (2008)^78^ USA	18^a^	na	F27.78 M72.22	Na	37.22 *(14.55)*	28.4 *(6.3)*	nr	Diagnosed	Consultation, Researcher, Author	Telephone; personal contacts
Tierney et al. (2016)^60^ UK	10^b^	na	F100.00	Na	14.40 *(1.07)*	na	nr	Diagnosed	Pre-screening questions with nonparticipants, Respondent validation	Purpose sample identified through CAMHS and self-referral to ASD charities
Hull et al. (2017)^59^ UK	92	na	F59.78 M32.61 O7.61	Na	43.15 *(4.23)*	na	Assumed by ability to complete the online survey	Diagnosed	Consultation, Respondent validation	Cambridge Autism Research Database; social media
Hickey et al. (2018)^61^ UK	13	na	F23.08 M76.92	Na	60.38 *(5.91)*	na	nr	Diagnosed AQ	Consultation	Leaflet from NHS; autism support groups, London
Schneid and Raz (2020)^79^ ISRAEL	24^c^ 22A 2SD	na	F54.27 M41.67 O4.16	na	31.00 [16–31]	na	na	Diagnosed/Self-Diagnosed	Researcher	Personal acquaintance; snowball
Crompton et al. (2020)^80^ UK	12	na	F83.33 M16.67	na	33.58 *(10.06)*	na	WASI-II	AQ	Consultation, Researcher	Social media; project website; autism organizations
Bradley et al. (2021)^66^ UK	277^d^	na	F66.43 M33.57	na	38.58 *(2.31)*	na	na	Diagnosed AQ	Design, recruitment, dissemination findings	Autism research center; online adverts
Halsall et al. (2021)^44^ UK	8±	na	F100.00	na	13y 7 m *(11.17m)*	na	WASI-II	Diagnosed SCQ	Design	Purpose sample of autism research base attached to mainstream school

^a^ Includes two informal autism diagnoses by medical doctor or mental health professional.

^b^ Six participants accompanied by parent.

^c^ Includes a 16-year-old participant.

^d^ Plus eight mothers and seven educators, no demographic information provided.

nr, not reported; na, not applicable; F, female; M, male; O, other; *M*, mean; NA, nonautistic; A, Autism; SD, self-diagnosed autistic; r, range; AU, Australia; USA, United States of America; UK, United Kingdom; CAMHS, Child and Adolescent Mental Health Service; NHS, National Health Service United Kingdom; Cog, cognitive ability; WASI-II, Weschler Abbreviated Scale of Intelligence; AQ, Autism Quotient; SCQ, Social Communication Questionnaire; y, years; m, months.

### Study quality

The quality of the included studies was mixed. Seven of the eight studies gave a clear statement of the aims of the research, one study was unclear.^80^ All included studies sought to learn more about the subjective experiences or autistic individuals; thus, the use of qualitative methodology was considered appropriate. Most studies were assessed as meeting each of the criteria satisfactorily, although some studies were lacking in quality. Five of the studies included community participation in the development of the study methodology, three of which included autistic authors.^78–80^ Crompton et al. did not clearly justify the research design, recruitment strategy, or the data collection method, and although the data analysis was considered rigorous, there was not a clear statement of findings that was contextualized within the broader literature.^80^ It was difficult to tell whether Müller et al. clearly justified the use of the recruitment strategy or data collection methods, considered ethical issues, or whether the data analysis was sufficiently rigorous.^78^ Crompton et al. research aim was unclear, which meant it was difficult to tell whether their findings answered the aim of the study.^80^ Four studies demonstrated detailed data analysis, including many participant quotes, and reported clear and well contextualized findings within the existing literature.^44,59,66,79^ The other study results were more interpretive, and the discussions were mostly often repetitious, rather than contextualized within the existing literature. The methodology applied by Bradley et al.^66^ meant that only participants who endorsed camouflage use responded to questions related to these strategies, which may have biased results.^66^ See Supplementary Data S1 for more details of study quality based on the CASP tool.

### Participant demographics

The participant and study demographics are displayed in Table 2. Overall, 454 participants contributed data to the eight studies. Of these, 450 reported a formal diagnosis of autism, two reported an informal diagnosis of Asperger’s Disorder (by a medical doctor or healthcare professional), and two individuals self-identified as autistic from one study, which was included due to representing only <10% of the study sample. All studies used a nonrandomized purposive recruitment strategy seeking autistic adolescents and/or adults. Halsall et al. interviewed eight mothers and seven educators in addition to autistic participants, whereas 6 out of 10 participants in Tierney et al. were accompanied by a parent at interview.^44,60^ Overall, the participants of included studies were convenience samples, and participants were recruited from clinical and school environments through advertising flyers, social media, and word of mouth.

The nationality of participants was reported in only two studies, and most participants were from Western countries, including Australia, United Kingdom, and United States of America.^44,59^ Most of the participants, were currently enrolled in mainstream education, or enrolled in a special education unit attached to a mainstream school, and some were home schooled.^44,60^ Two studies did not report on participant education or employment status.^52,80^ All participants in Müller et al. were either in school at the time of data collection or had previously been in higher education.^78^ Most of the remaining studies that reported on education and employment status found that most participants were either employed, volunteering, or currently studying.^66,79^ Participants in Hickey et al. were either unemployed (38%), or had retired (38%), and only 17% were employed at the time of data collection.^44^

Six studies recruited adults only (18-years-old and above) and two recruited only adolescents. Sample age ranged from 12 to 65 years of age. Sex distribution favored females (64.30%), and the remaining participants either identified as male (34.23%) or nonbinary (1.47%). Two studies recruited females only, and only two studies reported nonbinary participants. Sample size varied from eight autistic adolescents to 277 autistic adults. In three studies, autistic identity was self-reported by adult participants, and in two studies the parents of adolescent children reported a formal autism diagnosis. In three studies, adult participant diagnosis was confirmed using the Autism Quotient, and in one adolescent study autism diagnosis was confirmed using the Social Communication Questionnaire.^81,82^ Only two studies used a cognitive measure to confirm cognitive ability (see Table 2).

### Qualitative themes

Twenty-nine cumulative main themes emerged from the eight studies. Intimate relationships were rarely mentioned, as such, our themes relate mostly to friendship. A meta-thematic analysis of study themes that pertained to the aims of this review revealed three meta-themes. These were (1) Effectiveness of Camouflage, which captured how useful camouflaging was in facilitating desired friendships and intimate relationships; (2) Inefficiencies of Camouflage, which reports on the disproportionate consequences of camouflaging over social gains; and (3) Alternatives to Camouflaging, which describes mechanisms that facilitate desired social connection without relying on camouflaging. Individual study themes are presented in Table 3, and results of the meta-thematic analysis are described below.

**Table 3.** Data Collection Method and Identified Themes for Included Studies

Author and date of publication	Data collection method	Themes identified
Müller et al. (2008)	Semi-structured interview	1) Social experiences; Lack of social skills; Longing for intimacy; Desire to contribute to social interactions; Compensatory behaviour of increased social/self-awareness to improve social skills/social cognitions and increase social interaction 2) Social Support; Joint focus and shared interest activities to allow for social opportunities; Parallel activities allow for feeling connected without having to interact; structured social activities allow for interaction; enjoy community participation with little social interaction to feel involved; high predictability in social structure; prefer one-to-one or small groups help enable positive social interaction; small groups are a chance to observe, learn, and increased connection; prefer a group leader to help facilitate the group; appreciate facilitated social interactions; observing, learning, and imitating other people to improve social ability; communication support—find alternative methods of communication helpful (phone, internet) and will make behaviour changes to fit in; social skills useful to improve future ability to engage with others; suggested ways NAs could better befriend and support ASDs
Tierney et al. (2016)	Semi-structured interview	1) Experiences of social environment; Incommodious; Impenetrable; Misunderstandings and Mislabelings 2) Desire for friendships; Context for befriending; Challenges to developing friendships; Motivations for befriending 3) Overcoming challenges; External support; Innate skills; Imitation; Masking; Repercussions of strategies 4) Developing tasks; Triggers of difficulties; Sexual identity
Hull et al. (2017)^a^	Online open-ended question asking participants to leave comments on camouflaging	1) Assimilation (49F; 20M; 7NB); a question of safety; functioning member of society; normal enough 2) To know and be known (42F; 24M 5NB); opportunity to connect; to reduce a sense of stress 3) Compensation (45F; 22M; 7NB); the right kind of expressions; every single conversation plotted out 4) Masking (38F; 18M; 7NB); avoid being ‘too autistic’; play the appropriate role 5) People have a stereotyped view (32F; 6M; 4NB); you can’t be autistic; too good at masking the difficulties 6) I fall to pieces (44F; 21M; 7NB); drains my energy; I worry about getting it wrong 7) I’m not my true self (31F; 16M; 3NB); who the Hell am I?; betraying myself; deceiving people
Hickey et al. (2018)^a^	Semi-structured interview	1) Difference; realization of the difference (11); reducing outward difference (6) 2) Life review; understanding the past (13); externalising autism (8); self-acceptance (5) 3) Longing for connection; isolation and loneliness (9); positive aspects of isolation (6); reaching out pre-diagnosis (12); reach out post-diagnosis (10)
Schneid & Raz (2020)	Semi-structured interview (phone, email, face-to-face)	1) ‘Passing as normal’: impression management as social asset 2) ‘What is wrong with me?’: the ambivalence of impression management and camouflaging 3) The limits of impression management 4) An alternative to camouflaging? Autistic forms of social communication
Crompton et al. (2020)	Semi-structured interview	1) Across neurotype difficulties; within neurotype ease 2) Majority social norms; majority social activities and contexts; the impact of being a minority 3) Understanding; being your authentic self; happiness, well-being, and resilience
Bradley et al. (2021)	Open-ended questions in an online survey	1) Dangers of camouflaging; it’s exhausting; leads to ill mental health; others not accepting of my autistic self; creates unreal perceptions and expectations 2) Positive aspects of camouflaging; allows me to access the social world; thriving or surviving?; protective and resilience building 3) Why I don’t need to camouflage like I used to; not since being diagnosed; not when I’m understood and accepted
Halsall et al. (2021)	Semi-structured interviews with girls, parents, and educators	1) Inconsistencies, Contradictions, and Conflicts in Attempts to Camouflage; Self-developed and Adult-Led Camouflaging Strategies; Attempts to Camouflage Immature Interests; Different Camouflaging in Different Contexts; Embracing Autistic Identity, Resulting in Inconsistencies in Camouflaging 2) Using Camouflaging to Overcome Challenges in Masking and Maintaining Friends; No Girl’s Land. Unsuccessful Camouflaging Resulting in the Girls Not Fitting in Anywhere; Compatible Camouflaging Demands in Friendships with SEN pupils; Seeking Safe and Controllable Relationships to Reduce Camouflaging Demands 3) Camouflaging Learning Needs and the Challenges of Learning Inclusion; ‘She Doesn’t Like Feeling Stupid’. The Desire to Camouflage Learning Challenges; Dependence on Adults to Camouflage Challenges in Learning and School Life; ‘That’s Her Safe Space’. The Benefits of the Resource Base 4) Consequences of Camouflaging on Social Interaction, Learning, and Mental Health; Late Diagnosis and Missed Opportunities Due to Camouflaging; Immediate Impacts of Camouflaging (Exhaustion, Anxiety, and Emotional Distress)

^a^ Parenthesis show number of participants that nominated theme.

F, female; M, male; NB, non-binary.

#### Effectiveness of camouflage

Camouflaging helped to facilitate friendships and intimate relationships.^66^ Friendships were described as having someone to spend time with, either as a companion for sharing an interest, someone to relate with over similar life experiences, and feeling close to other people. Some autistic individuals found that camouflaging was initially necessary to obtain these types of relationships, although they were often not as close or meaningful as the individual desired. This was because the individual felt that they had to stay camouflaged as they experienced rejection when they revealed their true social selves.^66^ From adolescence, camouflaging was reinforced by parents and educators who often prompted and taught expected social communication and interaction skills.^44,79^ Early attempts at camouflaging for autistic adolescents were intermittent and unsophisticated, but these developed as adolescents began to better understand and mimic complex social behavior, and generalized their camouflage strategies.^44,59,61^ Despite similar camouflaging strategies and motivations, autistic individuals varied in camouflage complexity, success, and failure in its utility to help gain social acceptance and friendship.^44,60,61,78^ Most individuals camouflaged intensely when among the general population, where its use was necessary for access to the social world.^61,66^ The more successful autistic individuals were at camouflaging, the less recognizable their social difficulties were to those around them, which increased social pressure to conform and maintain camouflage.^57^ Camouflaging challenged their identity and felt like a personal betrayal, which effected how meaningfully they connected with others.^59^

Camouflaging was for many autistic individuals a conflictual experience, yet autistic individuals continued to rely on it in most social interactions.^59,61^ Some considered camouflaging as a necessary strategy, like that used by non-autistic individuals in various social situations; yet regardless of the effort applied to camouflaging, for most it became more difficult to sustain, and socializing became increasingly challenging with age.^59,79^ Other autistic individuals felt that camouflaging became easier with age, as they grew familiar with social expectations, and developed increasingly effective compensatory strategies that became less arduous overtime and more habitual.^61^ Regardless, participants reported an overall dissatisfaction with their friendships despite intense camouflaging, and in many cases, they did not foresee that this would likely improve.

#### Inefficiencies of camouflage

For many autistic individuals, camouflaging did not help them to achieve favorable social outcomes.^59,78^ Autistic participants expressed the perception that maintaining social status required them to either continue to camouflage or risk rejection; therefore, camouflaging felt coercive, and was stressful, exhausting, draining, and inauthentic.^59,60,79,80^ Some autistic individuals felt it was only possible to be authentic when interacting with peers who also had difficulties socializing, younger children, or family members, especially those who were also autistic; where camouflaging usually felt less necessary.^44,60,66,80^

The awareness of missed social opportunities and of attempts at communication being misinterpreted by others was distressing for most autistic individuals, particularly given the effort employed to build and maintain relationships.^78^ Most autistic individuals lacked reciprocal, authentic friendships, and felt socially isolated and lonely.^44,59,61,78^ Unsurprisingly, autistic individuals continued to desire deeper friendships, more friendships, and intimate relationships.^78^ Those who felt resigned to accept having their social desires dissatisfied, described embracing isolation; however, this resolve commonly led to intense loneliness, which contributed to anxiety and depression.^61,78^ Autistic adults who felt camouflaging enhanced their ability to interact viewed the strategy more positively, but described their social interactions and relationships as distant, unsatisfying, unsustainable, and lacking in genuine connection.^59,61^ Simply fitting in with others was described as enough for some, who felt their camouflaging skills were proficient, worthwhile, and led to improved quality of life with age.^61^ Others felt that the most authentic social relationships that they developed and maintained in the absence of camouflaging, although this prospect was uncommon as innate social behaviors, were usually less accepted by others.^61^

#### Alternatives to camouflaging

Although, camouflaging was the most common socialization strategy used by the autistic individuals to gain friendships, it was typically the least preferrable.^59,61,66,78–80^ However, many felt that they relied on camouflaging where they felt that there was no available alternative strategy to help connect with others.^44^ Camouflaging was so proficient for some that the severity of their actual challenges were often invisible to others, and therefore those who disclosed their autism diagnosis were often met with skepticism, which was distressing for the autistic individual.^59^ Often, in response to the negative consequences of camouflaging, some autistic individuals chose to minimize social participation and to spend time alone engaged in solitary interests.^61,78^ While this strategy offered camouflage reprieve, it also reduced the frequency of social interactions necessary to build friendships.^78^ Other autistic individuals opted for alternatives, including written communication or using objects as props to support conversation, yet these strategies were insufficient and ineffective in most social interactions.^78^ With age, autistic individuals become better at identifying factors that could support improved social interactions beyond camouflaging, including activity types, such as those that are joint focused or goal orientated, structured, facilitated, predictable, small, or one-on-one, and minimally affected sensory sensitivities.^59,78^ Other factors, related to the ideal qualities of others, included empathy, shared interests, leadership, guidance, autism awareness, like-mindedness, and feeling accepted by others; unfortunately, these mechanisms also appeared to be associated with decreased social opportunities, which limited some participants’ chance to build friendships and/or intimate relationships.^61^ Self-acceptance and self-compassion were considered important to reduce the internal pressure to skillfully navigate social environments.^61^

The acquisition of an autism diagnosis offered many autistic adults a context in which to better understand their social difficulties, as well as to reframe their identity and social history, and reduce reliance on camouflaging.^61,79^ Socializing in autistic communities offered some adult respondents the greatest opportunity to minimize, or cease, camouflaging and obtain friendship.^66,79,80^ Yet, identifying with and socializing in the autistic community also challenged the identity of many autistic individuals. Some perceived themselves as unlike other autistic people and chose to distance themselves from their own autistic characteristics and the autistic community, which increased their reliance on camouflaging.^61^ Family members, regardless of autism status, offered acceptance and reprieve from camouflaging for many respondents.^66,80^ In contrast, others reported that dropping camouflaging resulted in the loss of important relationships, including marital partners.^66^

## Discussion

The current study systematically reviewed existing literature on the friendships and intimate relationships achieved through camouflaging by autistic individuals. Little reference was made to intimate relationships in the literature; thus, the results and our interpretation relate mostly to friendship. We found a lack of quantifiable evidence about friendships and intimate relationships that are obtained when camouflaging, which is surprising in the context of the qualitative findings. Three meta-themes were revealed: (1) Effectiveness of Camouflage, (2) Inefficiencies of Camouflage, and (3) Alternatives to Camouflaging. We found evidence from the eight included studies that most autistic participants related with friends and/or intimate partners differently to non-autistic people, yet the subjective desire to have meaningful, authentic relationships remained consistent with non-autistic populations,^83,84^ in contradiction with SMt. Regardless of age, we found that the trajectory of camouflage development and its effectiveness in building and maintaining friendships was a similar experience for all autistic participants, which demonstrated a lack of alternative options.

We found that camouflage was an overwhelmingly necessary mechanism for socialization and friendship development, which persisted life-long despite being exhausting and taxing; a survival strategy in a non-autistic world.^45,64^ Its importance was recognized most commonly between late childhood and early adolescence, and was typically reinforced by close family members, teachers, and broader society.^44,60^ Camouflaging developed rapidly, as autistic adolescents desperately attempted to meet social expectations and feel connected with their peers. Initially superficial, camouflaging became increasingly sophisticated, intense, and more complex with age, shaped by the social interactions that occurred overtime.^47,62–64^

Our results suggest that the effort required to camouflage most often outweigh the social benefits, meaning that for many autistic individuals, camouflaging is a minimally effective strategy, and inefficient to obtain friendship. This may be because camouflaging requires intense attention and energy,^85^ while also necessitating the restriction of self-regulatory behaviors that are often required to cope during stressful social situations, thus social interactions feel awkward and superficial.^86,87^ The coercion felt by the autistic individuals to begin and maintain camouflage, regardless of the personal implications, is likely a response to the stigmatization and discrimination that is associated with being misunderstood by non-autistic people, and a longing to be seen and to fit in.^48,59^

We also found that camouflaging contributes to decreased social participation and social connection for some individuals, despite its purpose being to enhance these social outcomes.^61,78^ The decision to limit socialization occurred when camouflaging became too taxing to maintain, especially where it had failed to help the individual to obtain their socially desired outcomes.^44^ These experiences likely affect the way autistic individuals relate with others and approach friendship, therefore, reduced social engagement is likely a consequence of social experience rather than a preference for the participants in this review. Those who chose to limit camouflage yet not to decrease socializing, actively attempted to improve social awareness,^78^ or engaged alternative strategies in attempt to interact and make friends, including written communication, and being selective about the social context in which they choose to engage.^61^ While these may be useful alternatives to camouflage, they emphasize the consistent responsibility felt by many autistic people to find ways to enhance social opportunities, and may be lesser known examples of camouflaging.^45,88^

Our results suggest that camouflaging is a barrier to building authentic, close, and meaningful friendship. We found substantial evidence that the social relationships obtained through camouflage were often perceived as less satisfying than anticipated.^44,59^ Furthermore, there was evidence that this problem may be due to the dissonance caused when trying to connect the authentic self through an artificial version: the artificial version achieves an artificial connection while the authentic self remains unknown, unvalidated, and unconnected.^59^ Camouflaging ultimately conceals one’s identity, thus not only are many autistic individuals presenting inauthentically, but they are also unintentionally reinforcing stigmatizing beliefs about autism.^15,69^ Our findings evidence that the friendships obtained through camouflage are often superficial and are likely at risk of ending, should camouflaging reduce or cease,^66^ or the strategies employed fail to meet the expectations of others. We suggest that to better understand the friendships of autistic individuals, it would be helpful to understand the contributions of non-autistic social partners in social interactions, and their perceptions of autistic individuals both when camouflaging and when interacting authentically.^89^

Some autistic individuals in this sample who are motivated to socialize and also identify positively with their autistic diagnosis, seek social connections within the autistic community, where camouflage seems less necessary.^60,66,78^ These findings suggest that having a positive autistic identity can facilitate social connections with likeminded individuals through autistic support networks.^90^ Although such autistic connections can improve well-being, they do not appear to protect against the negative effects on their well-being inflicted by the camouflaging that is felt necessary when engaged in daily social interactions with non-autistic individuals.^90^ Other autistic individuals felt unwilling to identify with the autistic community, especially where they felt that they differed in their autistic characteristics and individual interests to others in the community. The choice to isolate from others who share a stigmatized identity may signify internalized stigma,^91^ which is associated with reduced friendship satisfaction and well-being, and increased suicidality among autistic people.^6,92^ As such, those autistic individuals who are motivated to connect, may be less likely to feel they have a community with which they identify, and be more vulnerable to isolation, loneliness, reduced well-being, and poorer mental health outcomes.^6,20–24^

We found that a range of other strategies are used to obtain friendship and/or intimate relationships by autistic individuals who set out to achieve this endeavor. Through repeated social experiences, autistic individuals who feel able to persist, learn that there are other ways to access social connection. Examples are characteristics of the social environment, such as geographical accessibility, activity structure, interest-based, small groups, and environments with minimal sensory input.^17,93,94^ Other factors involve characteristics of the social partner; for example, empathy, autism awareness, understanding, and acceptance.^16,80^ These factors can reduce the pressure to camouflage and increase social participation, which may then increase one’s authenticity in social interactions and friendships. The success of these alternatives is reliant on non-autistic individuals embracing diverse communication styles and perceptions, which would help to relieve the overwhelming pressure on autistic individuals in social interactions.^95^ Such modifiable factors are being introduced, through autism awareness training in both workplaces and educational environments, in collaboration with a growing number of non-autistic autism allies.^95–97^ The beneficial outcomes found from implementing changes in these environments suggests their potential utility in other social environments to: (1) increase social participation, (2) facilitate authentic and more meaningful relationships, (3) support positive self-identity, and (4) produce fewer negative outcomes for autistic individuals.^18,98^ These changes will be most successful when autistic and nonautistic individuals collaborate from research through to implementation, necessitating that autistic strengths are recognized and harnessed.^9^

### Limitations of the Review

Camouflaging is a rapidly growing area of research, and it is possible that the search strategy used may not have captured all potential synonyms, and, therefore, our findings likely apply to a specific group of autistic people based on our study selection criteria. We did not find any studies that addressed the key terms of the review that did not meet our criteria, which may be a consequence of our search or a reflection of the literature available that addressed the aim of this review. The study aims, recruitment strategies, and data collection procedures engaged by the included studies likely attracted autistic individuals who use social media, as well as those with strong verbal communication and social skills. These autistic individuals were also more likely to be socially motivated and actively seeking social connection and may have better developed camouflage skills compared with other autistic people.

Included studies used convenience samples where autistic females were overrepresented, and no comparison group data, we were unable to examine population differences. Studies were conducted in Westernized countries, and the authors note that intersections, such as culture, country, disability, and geographic location, were mostly unreported included studies. Finally, we found that most participants were above18 years of age, and although commonalities in social and camouflaging experiences were found between autistic adolescents and adults, the authors recognize that there are fundamental developmental and generational differences between 12-year-olds and 65-year-old autistic people unaccounted for in the current review that likely affect the interpretation of our results. To date, the literature in this area remains disproportionately qualitative, thus it is difficult to draw conclusions and the review findings should be considered as exploratory.

Only half of the included studies involved an autistic researcher and author. Autistic community participation was engaged for study design, including in the development of interview questions, in seven studies. It is possible that participants’ responses and the interpretations of results were affected by a lack of community participation.

### Conclusion

Our findings support overwhelming evidence that blame for social disconnect weighs heavily on the autistic individual, and that to overcome these challenges requires persistent and determined efforts that consistently challenge their identity. Camouflage use in the autistic sample strongly supports the presence of social motivation in this population, and evidences that camouflage is necessary to reduce obvious social differences and connect with others lifelong. We found that the emotional and psychological costs of camouflage appear to outweigh the benefits, particularly when one considers the low levels of satisfaction expressed by participants about the quality and authenticity of the friendships they establish and maintain this way. Structured social opportunities using small groups and controlling sensory stimulation, engaging with individuals who are inclusive of difference, can provide a safe place for seeking friendship and intimate relationships that reduces the need for camouflage. In the absence of reciprocal interactions, autistic individuals who reject or fail with camouflage often remain isolated and lonely, and at an increased risk of adverse mental health outcomes. Many autistic individuals seek to be able to participate freely in any social environment, rather than feel restricted by autistic communities, where they can often feel they do not belong.

There appears to be a complex interaction between social motivation, camouflage, the authentic self, and social relationships, which may be ameliorated in part by changes to the social environment and the attitudes toward autism held by non-autistic social partners. We recommend that research focus on the intersection of autistic and non-autistic social interactions toward shared responsibility between neurotypes, reducing the pressure to camouflage for autistic people. It is anticipated that this will help support autistic individuals to improve their social competency without compromising their identity and help to build and maintain authentic and rewarding social relationships.

## Notes

^1^
In this article, identity-first language will be used as it is often preferred by many self-advocates in the autistic community.^5^,^6^ The term *autistic* will be used to describe those diagnosed with an autism spectrum disorder, and those without a known diagnosis of autism spectrum disorder will be referred to as *non-autistic*.
