# Supplementary material for: Camouflaging Autism in Pursuit of Friendship and Intimate Relationships: A Systematic Review
Source: Autism Adulthood. 2025 Oct 24;7(5):543–57. doi: 10.1089/aut.2023.0160 (PMC12670704; doi:10.1089/aut.2023.0160)
Supplement: Supplementary file 1 [file aut.2023.0160_supplementarymaterial.docx]

**Table.** Study Quality as Assessed on the Critical Appraisal Skills Program – Qualitative Checklist

| **Study** | **Q1** | **Q2** | **Q3** | **Q4^a^** | **Q5** | **Q6** | **Q7** | **Q8** | **Q9** | **Q10** |
| --- | --- | --- | --- | --- | --- | --- | --- | --- | --- | --- |
| Müller et al. (2008) | Y | Y  Interviews to gather insights of autistic people’s experiences. | Y | C/T  Diagnosed Autistic people. | Y  Autistic participation included. | Y  Interview development and delivery considered individual needs. | C/T  Ethics not mentioned. | C/T  Few quotes used to support interpretation. | N | Respondent validation used.  Findings and interpretation not contextualised within the broader literature. |
| Tierney et al. (2016) | Y | Y  Interviews to gather insights of autistic people’s experiences. | Y | C/T  Diagnosed Autistic people. | Y | Y  Interview development and delivery considered individual needs. | Y  Ethical approval obtained. | Y  Quotes used to support interpretation. | Y | Findings and interpretations were contextualised only in literature that was consistent with study findings.  Many quotes presented to support author interpretations. |
| Hull et al. (2017) | Y | Y  Interviews to gather insights of autistic people’s experiences. | Y | C/T  Diagnosed Autistic people. | Y | Y  Interview development and delivery considered individual needs. | Y  Ethical approval obtained. | Y  Quotes used to support interpretation. | Y | The findings and interpretations were contextualised within the broader literature. Findings were additive to the area of research. Recommendations for future research and supports provided. |
| Hickey et al. (2018) | Y | Y  Interviews to gather insights of autistic people’sexperiences. | Y | C/T  Diagnosed Autistic people. | C/T | Y  Interview development and delivery considered individual needs. | Y  Ethical approval obtained. | Y  Quotes used to support interpretation. | N | Respondent validation. The findings and interpretation were not contextualised within the broader literature. |
| Schneid & Raz (2020) | Y | Y  Interviews to gather insights of autistic people’s experiences. | Y | Y  Diagnosed Autistic people. | Y | Y  Interview development and delivery considered individual needs. | C/T  Ethics approval not detailed. | C/T  Few participants’ quoted. | Y | Findings and interpretations were contextualised within the existing literature, and outcomes were used to guide future research and to inform ways to improve sociability for autistic people. |
| Crompton et al. (2020) | N  Unclear aim or research question. | Y  Interviews to gather insights of autistic people’s experiences. | C/T  No clear aim available. | C/T  Diagnosed Autistic people. | Y | Y  Interview development and delivery considered individual needs. | Y  Ethical approval obtained. | Y  Quotes used to support interpretation. | C/T | The findings and interpretation were contextualised within consistent literature, there were no contrary perspectives discussed. Findings were additive to the area of research. |
| Bradley et al. (2021) | Y | Y  Interviews to gather insights of autistic people’s experiences. | Y | C/T  Diagnosed Autistic people. | Y | Y  Interview development and delivery considered individual needs. | Y  Ethical approval obtained. | Y  Details about number of participants endorsing themes, plus quotes included. | Y | Findings were integrated into previous literature, with some unexpected findings contextualised. |
| Halsall et al. (2021) | Y | Y  Interviews to gather insights of autistic people’s experiences. | Y | C/T  Diagnosed Autistic people. | Y | Y  Interview development and delivery considered individual needs. | Y  Ethical approval obtained. | Y  Quotes used to support interpretation. | Y | Findings are integrated into previous literature, and the multi-informant approach allowed for more in-depth discussion. Limitations are outlined with recommendations for further research. The authors recognised the limited generalisability of findings. |

| Abbreviations: Y,yes this was demonstrated in the study; N, no this was not demonstrated in the study; C/T, Cannot Tell if this was demonstrated in the study; Q1, Was there a clear statement of the aims of the research?; Q2, Is a qualitative methodology appropriate?; Q3, Was the research design appropriate to address the aims of the research?; Q4, Was the recruitment strategy appropriate to the aims of the research?; Q5, Was the data collected in a way that addressed the research issue?; Q6, Has the relationship between researcher and participants been adequately considered?; Q7, Have ethical issues been taken into consideration?; Q8, Was the data analysis sufficiently rigorous?; Q9, Is there a clear statement of findings?; Q10, How valuable is the research? | ^a^ only formally/informally diagnosed autistic participants recruited, this neglects a population of undiagnosed and self-diagnosed autistic people. |
| --- | --- |
